# Common Neural Recruitment across Diverse Sustained Attention Tasks

**DOI:** 10.1371/journal.pone.0049556

**Published:** 2012-11-20

**Authors:** Jessica A. Grahn, Tom Manly

**Affiliations:** 1 Brain and Mind Institute, University of Western Ontario, London, Ontario, Canada; 2 Medical Research Council Cognition and Brain Sciences Unit, Cambridge, Cambridgeshire, United Kingdom; 3 Department of Psychology, University of Western Ontario, London, Ontario, Canada; University Of Cambridge, United Kingdom

## Abstract

At one level “sustained attention” is simply a description of a task demand. It is often used, however, in reference to a putatively unitary capacity to remain engaged in tasks that are lengthy, dull, repetitive and/or characterised by long intervals between relevant events. Deficits in sustained attention have been reported in a range of clinical conditions. Despite this, there is paucity of well-controlled human functional imaging evidence about regions commonly recruited during diverse sustained attention tasks. Here, for the first time, we used functional magnetic resonance imaging (fMRI) to monitor brain activity patterns as healthy volunteers performed two sustained attention tasks. The first, widely used in clinical assessment, required participants to count tones separated by long unpredictable intervals. This was contrasted with a control counting condition in which tones were presented at a brisk, regular rate. The second task was the Sustained Attention to Response Test (SART) in which participants responded to sequentially presented digits with the exception of a nominated infrequent no-go target. In the control condition, no-go trials were explicitly absent, removing the requirement to maintain a readiness to withhold responses. Although there were distinct patterns of activation associated with each task relative to its control, activity common to both tasks was found in the bilateral inferior frontal operculum, anterior cingulate, and bilateral premotor cortex. Although some researchers argue for a specific role of the inferior frontal operculum in inhibition, our results are consistent with recent findings of a more general attentional role for this area. The maintenance of a goal directed stance in the absence of strong environmental facilitation is challenging and this may underpin the sensitivity of sustained attention tasks to functional difficulties in a range of clinical groups.

## Introduction

The achievement of many different sorts of goals requires attention to be sustained over a certain duration. Sometimes the term “sustained attention” is used with reference to relatively brief periods of engagement, for example, in contrast to switching attention. The term has more generally been used, however, in the context of tasks that are long, repetitive and often quite tedious. Macworth's classic Clock Test [1] is a good example. Volunteers monitored the movements of a rotating hand around an unmarked clock face. Over two hours the hand moved at one interval per second. At unpredictable points, on average once per 150 movements, the hand would jump two intervals rather than one. This was the signal to which participants responded. Mackworth's key interest was not in the overall level of performance but rather in how well initial levels of performance were maintained under these unstimulating circumstances.

The perception that such lengthy tests were impractical and problems with the potential insensitivity of the “performance drop index” (vigilance decrement) to transient lapses in attention led other researchers to develop different paradigms. Wilkins and colleagues, for example, asked participants to maintain a count of stimuli that were presented at either a fast or slow pace [2]. They found that patients with lesions to the right prefrontal cortex had particular difficulty with slow presentations that could not be attributed to difficulty in counting per se. Accordingly this was interpreted as a problem in maintaining attention over the unfilled intervals in this rather boring task.

Robertson et al. [3] developed a different measure, the Sustained Attention to Response Test (SART). In the SART, participants watched a random series of single digits presented at the rate of 1 per 1.15 seconds in the centre of a computer monitor. They were asked to respond with a single button push to each digit with the exception of 3. The capacity to withhold responses to some but not all instances of the no-go target was interpreted as reflecting lapsing attentional control over the response. In support of this argument, subsequent studies showed that error rates were related to the mean interval between no-go trials [4], were reduced by cues reorienting participants' attention to their responses [5] and were elevated during periods of ‘task unrelated thoughts’ [6].

Sustained attention tests of this sort have proved useful in predicting function and outcome across a range of clinical conditions. For example, sustained attention function measured shortly after stroke is a better predictor of motor recovery over the next two years than concurrent measures of motor function [7]. Similarly, non-spatial sustained attention test performance is a significant predictor of recovery from unilateral spatial neglect after stroke [8] and of spatial biases in children [9], [10], [11]. Performance on the SART is correlated with everyday cognitive slips in volunteers with traumatic brain injuries and in the healthy population [3], [12]. Performance on slow stimulus counting and SART-style sustained attention measures is particularly poor in children and adults diagnosed with attention deficit hyperactivity disorder (ADHD [13]). In other words, although the sustained attention tests are generally rather artificial measures, stripped of variety and inherent motivation, they appear to capture important aspects of relevance to function in everyday activities.

Whilst there is evidence from large samples that slow counting and SART-like measures of sustained attention indeed show stronger correlations with each other than with other attentionally demanding activities [13], suggestive of overlapping demands, there is currently little evidence demonstrating the recruitment of common neural systems during diverse sustained attention tasks. In an early functional imaging study of sustained attention, Pardo [14] used a single slow stimulus counting task similar to that of Wilkins [2]. They reported increased activity relative to a no-task baseline particularly in right prefrontal and right parietal cortex (this finding was replicated in fMRI by Lewin [15]). Whilst this was consistent with a number of neuropsychological findings, the lack of control for other aspects of the task, such as counting, make it difficult to interpret the findings as specific to sustained attention. Ikkai and colleagues [16] also used a slow, unpredictable, silent visual counting task as part of a paradigm contrasting the maintenance of attention with switches in attention during functional magnetic resonance imaging (fMRI). In contrast to Pardo, they found that maintenance of attention activated the bilateral precentral sulci (dorsal and lateral premotor cortex), posterior inferior frontal sulci (mid-dorsolateral frontal cortex), dorsal anterior cingulate and inferior parietal sulci. This study offered little evidence of a lateralized pattern for sustained attention per se, simply that activity tended to be greater in whichever hemisphere was contralateral to the visual target.

Neural correlates of measures similar to the Sustained Attention to Response Test have also been measured. Ogg and colleagues [17] used fMRI to examine activity during performance of a task in which participants had to make responses to frequent go trials and withhold responses on occasional no-go trials. The authors noted bilateral dorsal premotor and anterior cingulate activation as well as right-lateralized ventral frontal and parietal activation. However, the baseline condition was simple fixation, so it is difficult to disentangle activation patterns elicited by sustained attention from those elicited by motor and visual characteristics of the task. A more controlled study was conducted by O'Connor et al. [18], in which fMRI was used to measure activation during the SART versus a control task (identical to the SART except that participants responded to every digit hence little attention to their responses was needed as no response inhibitions were required). They found that during the SART relative to control, there was significantly greater activity in right prefrontal cortex and thalamus, and a subthreshold increase in right parietal cortex.

Activity in right prefrontal and parietal cortex has been linked to behavioural performance measures in healthy participants. Using a rapid visual information processing task, Lawrence and others [19] found that right frontal and parietal activity correlated with good task performance (increased target detection amongst a stream of rapidly presented visual stimuli). Better behavioural performance on the SART and other tasks requiring vigilant attention can be enhanced by presenting non-informative auditory arousing tones randomly during task performance [20], [21]. Interestingly, when alerting tones are presented, right frontal activation is abolished, but not right parietal activation [18]. Thus, the right inferior parietal cortex may be a common pathway for both endogenous and exogenous attentional routes, mediating routine, semi-automatic maintenance of sustained attention. The right dorsolateral prefrontal cortex may have a role in more endogenous maintenance of sustained attention [22].

Thus, taken together, the neuropsychological and neuroimaging work implicate right prefrontal and parietal areas in sustained attention [14], [15], [19], [22], [23]. However, bilateral premotor and anterior cingulate cortices may also play a role, and not all studies find evidence of right-sided lateralization [16], [24]. The mixed picture may arise from the hetereogeneity of tasks and control conditions, as well as the difficulty in finding a single task that places demands on sustained attention, but not other aspects known to recruit frontal and parietal areas (cognitive demand, working memory, selective attention, etc.). A solution to this problem is to use two different sustained attention tasks, where at least one does not require a confounding cognitive process, and examine the conjunction of each (relative to control conditions) [25]. Thus, although each test may require cognitive components in addition to sustained attention (e.g., simple rule maintenance and response inhibition in the SART [26], [27], [28], and counting in the tone task), sustained attention is the central component required in *both* tasks. This cognitive conjunction approach [25] enables us to isolate the common component (sustained attention) even though each individual task may have other cognitive demands. No study has yet, to our knowledge, looked for the regions of common recruitment associated with increased sustained attention demand in very different sustained attention tasks in the same participants. This was therefore the main aim of the current study. An added motivation for choosing these tasks was their brevity and sensitivity to clinical deficits and likelihood of everyday cognitive slips [3], [7], [8], [9], [10], [12], [13].

**Figure 1:**
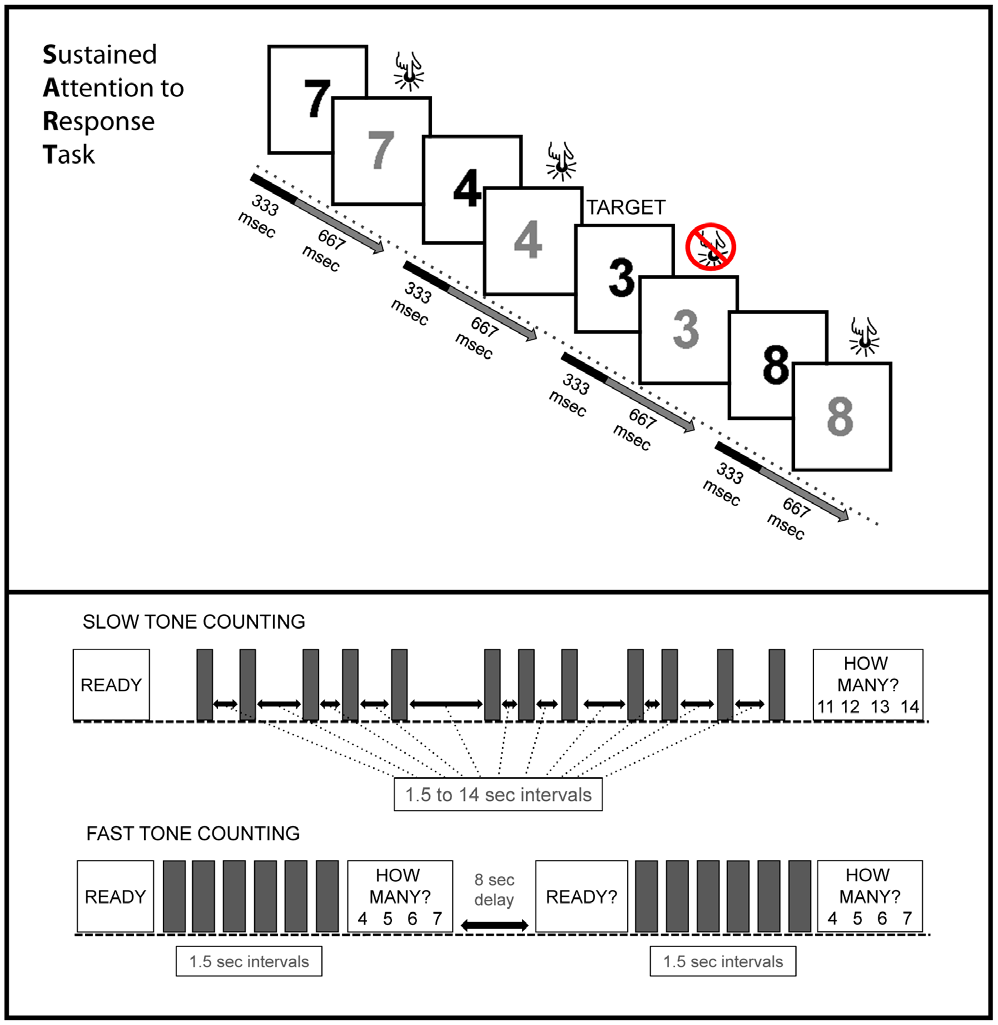
Schematic of the SART and the tone counting tasks. The top panel depicts the ‘hard SART’ condition, in which occasional no-go targets are presented. The ‘easy SART’ condition (not shown) is identical except that no targets are presented. The bottom panel depicts the slow and fast tone counting conditions (grey bars represent brief tones). A ready signal is followed by the auditory tones. The participants are then asked how many tones they counted. In slow tone counting, there are occasional long, unpredictable intervals between tones. In fast tone counting, the tones are presented every 1.5 seconds, but split into two groups separated by 8 seconds. Participants do not have to remember anything during the 8 second gap.

Here, participants performed two sustained attention tests during a single fMRI experiment. The first was a counting task in which the stimuli were separated by long and unpredictable intervals. Blood oxygen level dependent (BOLD) signals during this task were contrasted with those elicited during a control task in which participants counted brief runs of regular and more briskly presented stimuli interspersed with breaks (see Figure 1). Thus, the control condition contains the same stimuli and task requirements to count and respond, but with reduced demands on sustained attention. The participants also completed a standard version of the SART in which they were asked to make a button-push response to each presented digit with the exception of a nominated no-go target digit. This was contrasted with a control condition in which they again were asked to respond to all digits but were told, accurately, that no-go trials would not be presented. This differs from the O'Connor [18] SART control condition. In their control condition, stimuli designated as no-go trials in the SART were included, with participants now being asked to respond as if these were go stimuli. The removal of such trials minimizes the risk of residual no-go target processes occurring in the current control condition.

In terms of hypotheses there are a number of grounds on which to focus on the lateral prefrontal cortex. First, as discussed, there is a degree of concordance between the functional imaging and neuropsychological literature on the importance of the prefrontal cortex in sustaining attention [2], [14], [15], [24], [29]. Second, among the earliest findings from single cell recordings in lateral frontal regions was the discovery of neurons that fired during delays (e.g. between a target's location being signaled and the opportunity to respond to it); a form of sustained attention/memory/engagement [30], [31]. Third, one of the most striking findings from functional imaging over the last few decades has been how, in contrast with other brain regions that show rather specific responses (e.g. to certain classes of stimuli [32]), common lateral prefrontal regions are engaged in a very wide variety of attentionally demanding tasks [33], [34]. This apparent flexibility in function concords with more recent single cell recording work showing that lateral frontal cells change their response characteristics depending on the current task demands; a cells may switch from coding location to object identity as the task context changes [35] or may exhibit “delay activity” in one part of a trial and target identity in another [36]. As a result, these regions (together with the pre-SMA and anterior cingulate cortex, inferior frontal operculum, and inferior parietal cortex) have been argued to form a Multiple Demand Network that flexibly adapts to current task demands [33], [37]. In being implicated in the top-down establishment of goal-directed attention, the management of competing goals/task-sets more generally, and the maintenance of goal directed activity in the absence of current environmental triggers, the MD network is a prime candidate for increased activity to meet sustained attention demands.

On the other hand, premotor cortex has been implicated in some sustained attention tests [16], [17], thus an interesting question is whether it is engaged specifically by sustained attention or by other processes also present in previous tasks (shifting of attention, inhibitions of response, or target detection).

## Methods

### Participants

Eighteen right-handed neurologically normal volunteers (8 male) between the ages of 19 and 29 years (*M* = 22.9, *SD* = 3.5) participated. Informed consent was obtained from all participants. Participants completed two consecutive sessions of the tone counting and two consecutive sessions of the Sustained Attention to Response Task (SART) in a row. The order of the tasks was counterbalanced across participants.

## Materials and Design

### Tone counting

There were two conditions of interest in the tone counting experiment (participants also completed a third tone-counting condition that involved continuous tone presentation, but this condition is not relevant to the current investigation and will not be discussed further). In all conditions participants were asked to count the number of identical tones presented within a trial, with an instruction cue displayed for four seconds prior to the start of the tones. At the end of each trial they were asked to select via an appropriate button-push the correct total from a 4-choice array presented via a mirror suspended above their heads. During each trial the display was blank. The first condition was ‘slow’ tone counting, in which 12 to 18 tones were presented at inter-onset-intervals that varied between 1.5 to 14 seconds, with each trial lasting between 45 and 60 seconds. The second condition was ‘fast’ tone counting. Here, between 12 and 18 tones were presented, but divided into two groups with a constant inter-onset interval of 1.5 seconds between tones in each group. After the first group was presented, participants were presented with the 4-choice response screen. There then followed an 8 second delay before the onset of the second group. During this interval there was no need to maintain the previous total. The second group of tones was then presented, with participants again counting and then selecting the correct total from the 4-choice response screen [1]. Each fast tone counting trial was between 45 and 60 seconds. Thus, the number of tones and total amount of silence were identical between slow and fast tone counting, but fast tone counting reduced the need for sustained attention during the silent period. There was a 15-second inter-trial interval to allow estimation of a null baseline. In each session, there were three blocks, each block containing one trial of each condition. Trials were randomly ordered within a block. The two trial types are depicted in Figure 1.

Tones were presented diotically over headphones. Further attenuation of scanner noise was achieved with insert earplugs rated to attenuate by ∼30 dB (3 M 1100 earplugs, 3 M United Kingdom PLC, Bracknell, UK). When wearing earplugs and ear defenders, none of the participants reported difficulty in hearing the stimuli or focusing on the task. Participants were instructed not to move any part of their body during the scan (other than to respond). Button press responses were recorded with millisecond accuracy.

### Sustained Attention to Response Task

In each trial of the SART, a single digit (1–9) was presented in the centre of the mirror for 1 second. Each digit appeared with equal frequency in a pseudo-random sequence. The digit was displayed in black font for the first 333 ms, then gray font for the rest of the 1 second period. Participants were instructed to press a button under their index finger when each digit turned gray. This allowed participants to make responses at a steady pace of one per second, but ensured that they had time to view the number before a response was required. They were instructed to press the button for every digit except ‘3’. For 3's, they were to withhold the response.

The SART was divided into ‘easy’ and ‘hard’ blocks. During the ‘easy’ block, participants were correctly told that no 3's would be presented and therefore no responses would need to be withheld. In the ‘hard’ block, 3 s were presented at unpredictable intervals within the pseudo-random sequence. Each block was preceded by the relevant instruction and lasted for 30 seconds. In each of 2 scanning sessions, four easy blocks alternated with four hard blocks (each block was separated by 15 seconds rest). The order of block presentation (easy first or hard first) was counterbalanced across participants.

### Image acquisition

Participants were scanned on a 3T Siemens Tim Trio, using a head coil gradient set. Scanning of each volunteer was fully within the guidelines set out by the National Radiological Protection Board (“Principles for the Protection of Patients and Volunteers During Clinical Magnetic Resonance Diagnostic Procedures”, Documents of the NRPB, Volume 2(1), 1991). In every case, considerable care was taken to ensure that the volunteer remained comfortable throughout the session. The volunteers were positioned in the scanner with foam pads placed around the head and supporting the legs to ensure comfort and minimal movement.

Two SART sessions were conducted (∼6.5 minutes each), with 200 echoplanar imaging (EPI) volumes per session. Two Tone counting sessions were also conducted (∼11.7 minutes each), with 350 EPI volumes per session. EPI data parameters were: 32 slices, matrix size of 64×64, TE = 30 ms, TR = 2 s, FOV = 19.2×19.2 cm, flip angle = 78°. The resulting EPIs had a slice thickness of 3 mm, interslice distance of 0.75 mm, and in-plane resolution of 3×3 mm. High-resolution 1×1×1 mm modified driven equilibrium Fourier transform (MDEFT) anatomical images were collected for anatomic localization and coregistration.

#### Image processing and statistical analysis

SPM5 was used for data analysis (SPM5; Wellcome Department of Cognitive Neurology, London, UK). Images were slice-timing corrected, with the middle slice in each scan used as a reference. They were then realigned spatially (to correct for subject motion), with respect to the first image in the series, using trilinear interpolation. The MDEFT image was segmented and normalized (using affine and smoothly nonlinear transformations) to a brain template in Montreal Neurological Institute (MNI) space. The resulting normalization parameters were then applied to the EPIs and all normalized EPI images were spatially smoothed with an 8 mm full-width half-maximum Gaussian kernel.

#### SART analysis

For each participant, the blocks of easy and hard SART were modeled, in addition to events representing correct inhibitions (successfully withholding a response when a ‘3’ appeared), errors of commission (responding when a ‘3’ appeared), and omissions (digits other than ‘3’ were presented but no response was made). Each event was modeled using a regressor made from an on-off boxcar convolved with a canonical hemodynamic response function. For SART blocks, the duration of the boxcar equaled the duration of the blocks, and for trials representing inhibitions, commissions, and omissions, the duration was set to 0. Second-level analyses compared easy and hard block-related activity after modeling out inhibitions, omissions, and errors of commission.

#### Tone counting analysis

For each participant, each slow tone counting trial was modeled as a block from the beginning of the first tone to the offset of the last tone. The fast tone counting trials were modeled as a block from the beginning of the first tone of the first group to the offset of the last tone of the last group. Thus, both tone counting conditions contained equivalent numbers of tones and silence. Responses were modeled separately. Each trial was modeled using a regressor made from an on-off boxcar convolved with a canonical hemodynamic response function. The duration of the boxcar equaled the duration of entire tone-counting trial (see Figure 1), and for responses the duration was set to 0. Second-level analyses compared slow and fast tone counting blocks after modeling out responses.

For both SART and Tone counting whole-brain analyses, EPI volumes associated with discrete artifacts were included as covariates of no interest (nulling regressors). This included volume displacements >4 mm or spikes of high variance in which scaled volume to volume variance was 4 times greater than the mean variance of the run. Alternate analyses were conducted using 6 movement parameters as regressors of no interest and produced very similar results, therefore are not reported here. Autocorrelations were modeled using an AR(1) process and low-frequency noise was removed with a standard high-pass filter of 128 seconds. The contrast images estimated from single participant models were entered into second-level random effects analyses for group inference [38]. All whole-brain analyses were thresholded at pFDR<.05, whole-brain corrected [39].

To illustrate levels of activity within a specific brain region for the two sustained attention tasks, mean signal intensity was extracted from the relevant contrasts (hard-easy SART and slow-fast tone counting) using the software package MarsBar (http://marsbar.sourceforge.net). The regions of interest were defined from cortical clusters that were significant (pFDR<.05) in both the second-level random effects SART hard – easy contrast and the second-level slow – fast tone counting contrast.

## Results

### Behavioural results

In the tone counting task participants were significantly more accurate during the fast compared with slow paced task (fast accuracy = 93%; SD 12.0; slow accuracy = 81%; SD 22.0; t(1,17) = 3.49, p = .002). Participants ranged from 78% to 100% in correctly withholding responses on the hard SART, with a mean of 94% (SD = 6).

### Neuroimaging results

#### SART

**Figure 2:**
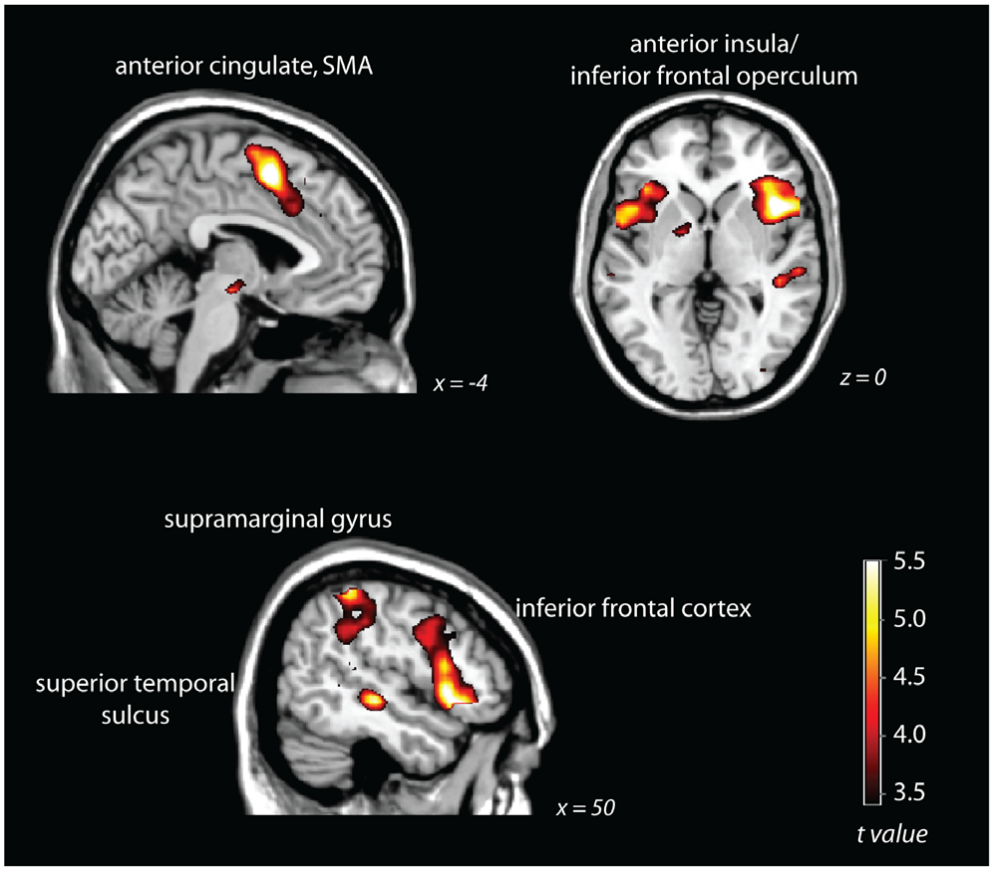
hard SART – easy SART contrast, thresholded at pFDR<.05, whole-brain corrected.

The contrast of hard SART blocks – easy SART blocks (after modelling targets and response inhibitions as regressors of no interest) revealed significant activity at whole-brain corrected levels (pFDR<.05) in the anterior cingulate cortex, bilateral inferior frontal operculum, right premotor cortex, and left premotor cortex (Figure 2). For details of maxima, see Table 1.

**Table 1:** Brain regions significantly activated in the hard SART – easy SART contrast.

Brain Area	Brodmann Area	cluster size	t value	p FDR	x	y	z
R supplementary motor area/anterior cingulate	BA 6/24/32	107	5.6	.019	9	9	48
L supplementary motor area/anterior cingulate	BA 6/24/32		5	.019	−9	12	48
R inferior frontal operculum	BA 47	41	4.52	.028	36	21	6
			4.29	.032	33	18	−6
L inferior frontal operculum	BA 47	29	5.07	.019	−27	24	3
R premotor cortex	BA 6	10	4.27	.032	30	0	48
L premotor cortex	BA 6	26	4.73	.025	−24	−6	63
			4.06	.042	−30	−6	54
Midbrain		27	5.18	.019	0	−30	−3

All reported peaks significant at p<0.05 whole-brain corrected (FDR) threshold.

#### Tone counting

**Figure 3:**
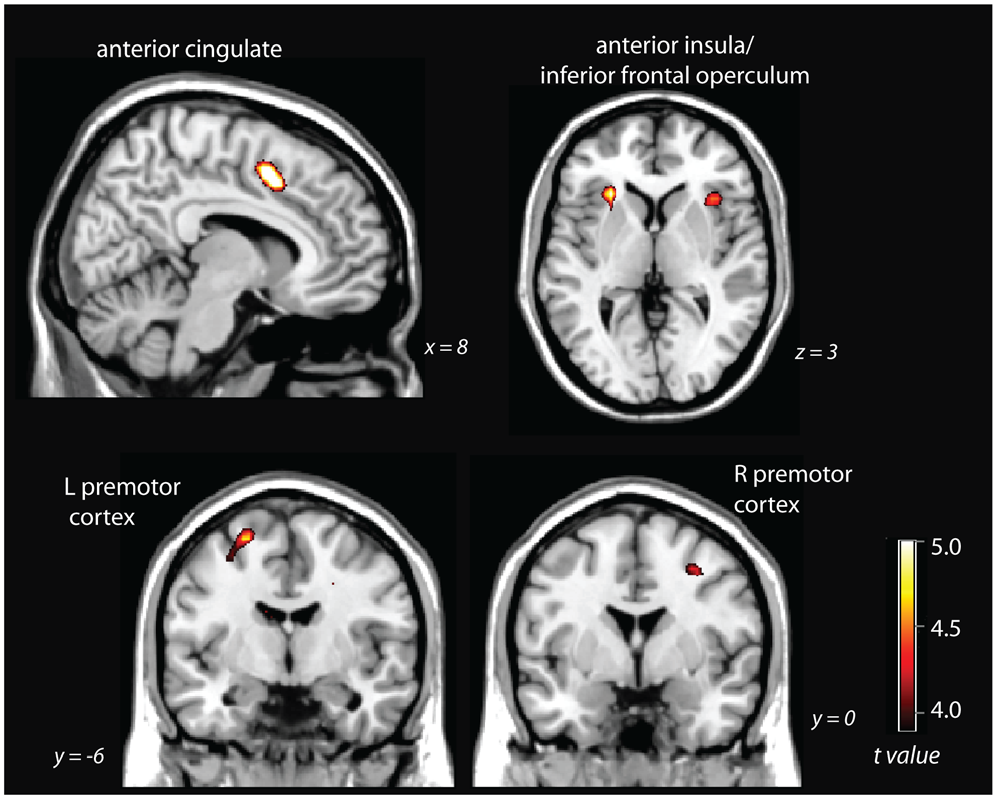
slow tone counting – fast tone counting, thresholded at pFDR<.05, whole-brain corrected.

The contrast of slow tone counting – fast continuous tone counting revealed significant activity at whole-brain corrected levels (pFDR<.05) in inferior frontal operculum, the anterior cingulate cortex, right premotor cortex, right supramarginal gyrus, and right superior temporal sulcus/middle temporal gyrus (Figure 3). For details of maxima, see Table 2.

**Table 2:** Brain regions significantly activated in the slow tone counting – fast tone counting contrast.

Brain Area	Brodmann Area	cluster size	t value	p FDR	x	y	z
R middle frontal gyrus	BA 46	†	4.36	.003	24	60	27
L middle frontal gyrus	BA 46	†	6.41	.001	−27	54	24
R supplementary motor area	BA 6	†	7.03	.001	12	6	60
L supplementary motor area	BA 6	†	6.68	.001	−6	6	51
R premotor cortex	BA 6	†	4.79	.001	45	6	39
L premotor cortex	BA 6	†	5.19	.001	−42	−3	48
L premotor cortex	BA 6	†	4.59	.002	−54	−3	45
L premotor cortex	BA 6	†	4.59	.002	−30	−9	57
R inferior frontal operculum		†	6.39	.001	30	18	−12
R inferior frontal operculum	BA 47	†	6.37	.001	45	15	0
R inferior frontal operculum	BA 47	†	5.7	.001	51	21	−6
R inferior frontal operculum	BA 47	†	5.58	.001	42	21	−12
L inferior frontal operculum		†	5.32	.001	−39	15	12
L inferior frontal operculum	BA 47	†	4.62	.002	−39	21	−3
L inferior frontal operculum	BA 47	†	4.94	.001	−33	27	6
R superior temporal gyrus	BA 42	†	5.26	.001	63	−42	18
L superior temporal pole	BA 38	†	5.05	.001	−48	15	−3
L superior temporal pole		†	4.9	.001	−54	6	0
R middle temporal gyrus	BA 21	†	6.65	.001	57	−24	−6
R middle temporal gyrus	BA 21	†	5.42	.001	45	−30	−3
L middle temporal gyrus	BA 22	308	4.53	.002	−51	−45	12
L middle temporal gyrus	BA 21	19	3.82	.006	−60	−27	0
R superior parietal lobule	BA 7	58	3.21	.015	21	−57	51
L superior parietal lobule	BA 7	16	2.91	.024	−18	−63	54
R inferior parietal lobule	BA 40	†	5.72	.001	48	−42	57
R supramarginal gyrus	BA 40	†	5.11	.001	60	−42	33
R inferior occipital gyrus	BA 19	142	3.6	.008	36	−87	0
R superior occipital gyrus	BA 18		3.49	.009	24	−96	15
L superior occipital gyrus	BA 17	48	3.16	.016	−15	−96	18
L middle occipital gyrus	BA 18		2.61	.038	−27	−90	3
Midbrain		†	4.92	.001	−15	−18	−15
Midbrain		†	4.9	.001	−9	−9	−6
Midbrain		†	4.65	.002	−3	−15	−12
cerebellar vermis		15	3.36	.011	0	−54	−27

All reported peaks significant at p<0.05 whole-brain corrected (FDR) threshold.

† = part of a cluster of 8779 voxels.

#### Overlap

**Figure 4:**
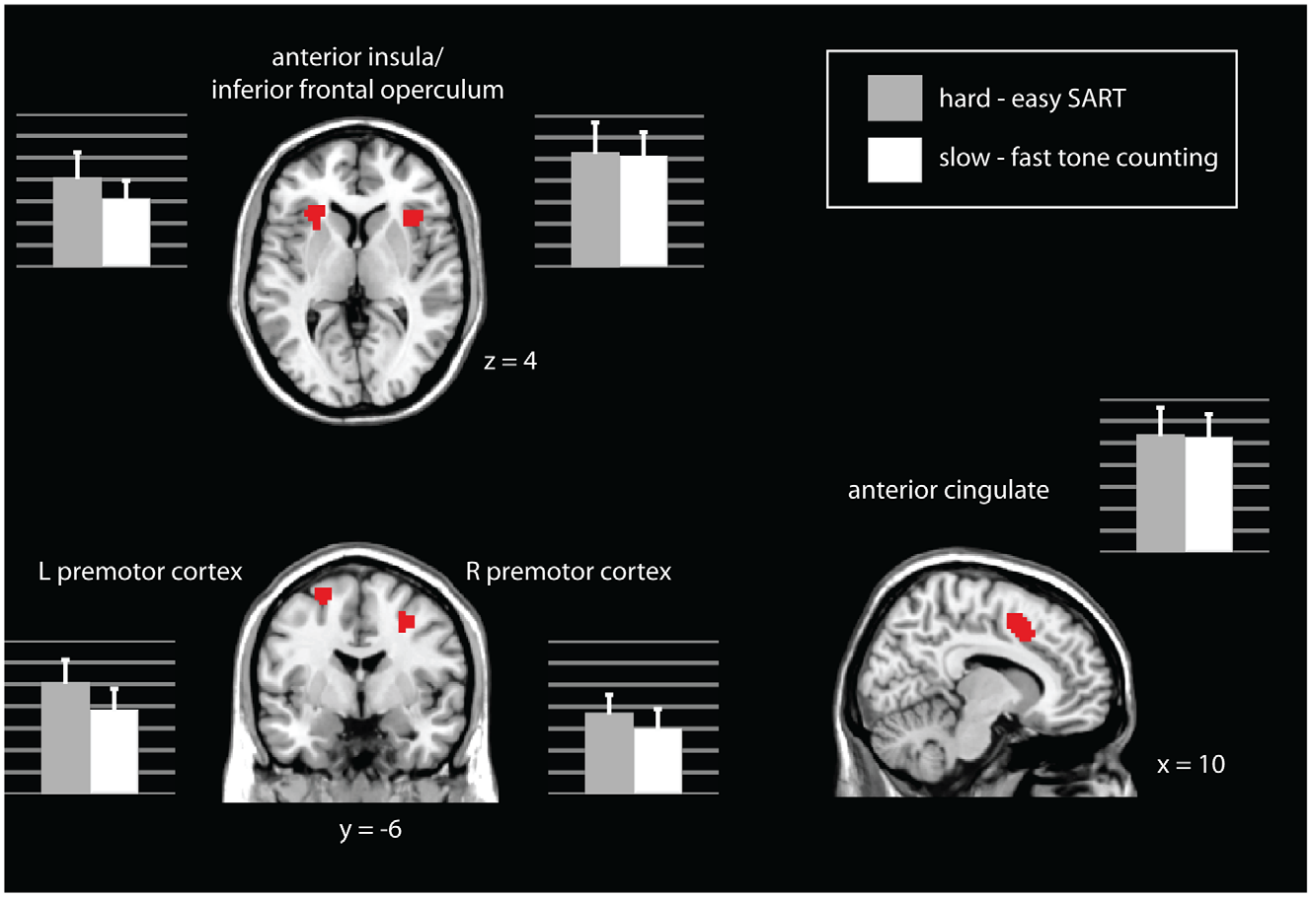
Areas in which both the hard – easy SART contrast and the slow – fast tone counting contrast were significantly active at pFDR<.05, whole-brain corrected.

There are several areas of overlap between the two contrasts. Using inclusive masking, the following areas were significantly active (pFDR<.05) in both the hard – easy SART contrast AND the slow – fast tone counting contrast: anterior cingulate cortex, bilateral inferior frontal operculum, and bilateral premotor cortex (as shown in Figure 4).

#### Laterality

To test for rightward lateralization in areas of homologous activation, the right and left inferior frontal operculum activity was compared for the hard - easy SART contrast and the slow - fast tone counting contrast. Paired t-tests showed that activity on the right compared to the left was marginally significantly greater for hard - easy SART (t(1,17) = 1.99, p = .06) and significantly greater for slow - fast tone counting (T(1,17) = 3.68, p = .002). Marsbar extractions of the parameter estimates for each condition in each region can be seen in Figure 4.

### Activation correlations with behavioural performance

To test whether activation differences in the regions of overlap correlated with behavioural performance, we took the accuracy for slow tone counting and hard SART performance for each subject, and correlated them with contrast estimates in the hard - easy SART and slow - fast tone counting conditions. No significant correlations were found between SART performance and activation the hard - easy SART task (all p's>.6), nor between SART performance and activation in the slow - fast tone counting contrast (all p's >.2). This may be due to the overall very high performance (94%) and restricted variability in the SART behavioural data. No significant correlations were found for tone counting performance and activation in the slow - fast tone counting contrast (all p's>.4). However, significant correlations were found between tone counting performance and activation in the overlapping brain regions for the hard - easy SART contrast (Anterior Cingulate: r = .71, p<.001; R inferior frontal operculum: r = .50, p = .035; L inferior frontal operculum: r = .62, p = .006; R premotor cortex: r = .55, p = .02; L premotor cortex: r = .73, p = .001).

## Discussion

In this study we found that a subset of brain areas were activated by each of two entirely different sustained attention tasks (relative to carefully designed control conditions), providing novel evidence for a generalized supramodal sustained attention network. At a whole-brain-corrected level of significance, increases in activity were observed for both the slow tone counting and SART measures compared with their respective controls in bilateral inferior frontal operculum, anterior cingulate, and bilateral premotor cortex. Crucially, the overlap was observed even though the tasks differed markedly in characteristics such as modality of input, requirement to count or withhold responses, the rate of stimulus presentation, and the frequency of responding. Moreover, behavioural performance on the slow tone counting task was significantly correlated with the degree of activation in all the aforementioned regions for the hard - easy SART contrast. Although it was somewhat puzzling that the behavioural and activation correlations did not reach significance for the slow - fast tone counting contrast, null effects are difficult to interpret, and it may be that the difference between slow and fast tone counting simply was not as variable across participants.

There was some evidence of a right lateralised activation pattern for sustained attention. Although right and left inferior frontal opercula were active, the amount of activation was significantly higher in the right than the left. Neuropsychological studies [24] have shown that right-hemisphere damage markedly impairs slow tone-counting performance, whereas left-hemisphere damage does not produce such deficits. Other neuroimaging studies have shown lateralized activation patterns for sustained attention [18], [23], [40]. The presence of significant left hemisphere activity may indicate that, although right-hemispheric structures are crucial for normal performance on sustained attention tasks, homologous left-hemispheric structures might also have some capacity for sustaining attention. The mixed neuroimaging picture is likely due to the variety of tasks used to measure sustained attention. Many previous studies use tasks that involve other cognitive processes known to activate right-lateralized frontal structures. For example, a recent study found that lapses in attention are preceded by decreases in right middle and inferior frontal gyrus activation, potentially implicating these structures in maintenance of sustained attention. However, the task involved *selective* attention to potentially interfering dimensions of global/local stimuli [41], which also required a substantial degree of cognitive control. Working memory is another common element in sustained attention tasks (and indeed was an element of one, but not both, of our tasks). Finally, extremely rapid stimulus presentation rates [19], or other factors that increase cognitive demand, may engage right-lateralized cognitive control regions.

Early reviews of the literature (e.g., Cabeza & Nyberg, [42]) do not generally report inferior frontal operculum activation for sustained attention tasks, instead focusing on more dorsal frontal areas such as Brodmann area 9/46 and 6 (premotor cortex). One debate in the literature is whether the inferior frontal operculum, particularly in the right hemisphere, is activated specifically by response inhibition [43] rather than having a more general role in sustained attention and processing of task relevant information [44], [45]. Our findings support the latter view: although the SART requires inhibition to infrequent no-go events, the tone counting task has no obvious inhibitory component, but the analyses indicated that the inferior frontal operculum was significantly activated in both tasks. Moreover, the activation was correlated with behavioural performance on the tone counting task, further supporting the interpretation of their role in sustained attention rather than inhibition in the current study. These findings are consistent with other recent work that has implicated inferior frontal areas in sustained attention in tasks without inhibitory requirements [46]. For example, activity in inferior frontal operculum increases parametrically with the duration of silence experienced when continuously monitoring for an auditory tone [47] . In addition, reductions in right inferior frontal activity are correlated with errors in the Continuous Performance Test, a test of sustained attention that involves responding to infrequent targets and does not have a response inhibition component [48].

The region of interest analysis suggests that the activity common to both sustained attention measures fell within regions known to be active across a range of attentionally demanding tasks: so-called Multiple Demand areas [33], [37]. In particular, the inferior frontal operculum and anterior cingulate cortex are commonly recruited as part of the MD network [33]. In accounting for this ubiquitous role, and taking into account a range of findings from single cell recordings in these regions [35], [36], it has been proposed that Multiple Demand areas flexibly adapt to the individual's current intentions/task to produce sequential goal-directed behaviour. In many circumstances we are faced with competing incompatible goals (reading this paper is in competition with useful chores on your to-do list, checking facebook, daydreaming, sleep etc). In this respect, the characteristics of sustained attention tests in which a participant must persist in maintaining a continuous task-directed stance *despite* minimal stimulation for that stance from the task and little inherent reward in doing so can be seen as a particularly strong challenge to the MD system. Their sensitivity to capacity limits of this sort may be why sustained attention tests appear sensitive to a range of everyday difficulties in clinical and healthy populations [2], [3], [49], [50].

An appropriate level of alertness (generalized wakefulness and responsivity) is a prerequisite for adequate sustained attention e.g. [51]. An aspect of the MD activity seen during sustained attention tasks may therefore be a form of endogenous activation to compensate for low levels of exogenous stimulation from the tasks. In this respect, connections between lateral prefrontal areas and the anterior cingulate and thalamic (intralaminar nucleus) and midbrain (reticular formation) regions engaged in alertness/physiological arousal may be relevant [52], [53], [54], [55].

It must be noted that only part of the MD network appeared to respond to the sustained attention requirements in our tasks. The tone counting and SART contrasts revealed increased activity in bilateral premotor cortex (Brodmann area 6), which is not typically included in the MD network, but has been activated in other studies of sustained attention [14], [56]. Influential views regarding the highly selective nature of attention argue that this allows coherent *action* and the premotor cortex peaks in our study are near the coordinates given by Corbetta et al. [40] as part of the bilateral dorsal attention network closely linked with action. The nature of these links across two sustained attention tasks that are very different in their ostensive motor requirements remains, however, obscure. One general possibility is that it reflects a form of vigilant preparedness to respond (or resist responding to potentially distracting stimuli). Another is that it reflects strategic inner speech used to compensate for minimal task stimulation (“the total is 4, listen out for the next tone” “don't get carried away, the next trial may be a no-go”). The link may be more abstract however. For example, evidence from single-cell recordings that the premotor cortex can code response ‘rules’ in much the same way that prefrontal cortex does (indeed, earlier and more strongly than prefrontal areas in some cases [57], [58]). Therefore, although the premotor cortex commonly appears in sustained attention tasks, further evidence is required to fully interpret whether it is the result of attention to action or encoding of rules.

In summary, ‘sustained attention’ is often considered as a unitary construct, with deficits in this capacity being seen as relevant to a range of developmental and neurological clinical conditions. Despite this, no study has to date directly examined overlap in neural activity between two sustained attention tasks that differ considerably in their surface characteristics. Here, using the SART and a slow tone counting measure, with careful controls for the peripheral aspects of each, we found regions of common bilateral activation: inferior frontal operculum, anterior cingulate, and premotor cortex associated with increased sustained attention demand. The results are consistent with the engagement of a subset of MD regions as well as premotor cortex in the effortful maintenance of attention to an unstimulating goal.
